# Shear wave elastography measurements in dogs treated surgically for congenital extrahepatic portosystemic shunts

**DOI:** 10.3389/fvets.2022.991148

**Published:** 2022-09-26

**Authors:** Merle Toom, Jimmy H. Saunders, Luc Duchateau, Goncalo Serrano, Hilde De Rooster, Nausikaa Devriendt, Emmelie Stock

**Affiliations:** ^1^Department of Morphology, Imaging, Orthopedics, Rehabilitation and Nutrition, Faculty of Veterinary Medicine, Merelbeke, Belgium; ^2^Department of Veterinary and Biosciences, Faculty of Veterinary Medicine, Merelbeke, Belgium; ^3^Department of Small Animals, Faculty of Veterinary Medicine, Merelbeke, Belgium

**Keywords:** elastography, 2D SWE, liver, dog, portosystemic shunt

## Abstract

Assessing the postoperative surgical success of congenital extrahepatic portosystemic shunt (EHPSS) attenuation can be challenging and involve invasive imaging methods. Elastography is an ultrasound technique that allows qualitative and quantitative estimation of tissue stiffness and has extensively been used in people with liver disease. In recent years, increased interest in this technique has developed in veterinary medicine due to its non-invasive nature, availability, and low cost. The objective of this study was to compare liver stiffness values between dogs with closed EHPSS and those with multiple acquired portosystemic shunts (MAPSS) after gradual surgical attenuation and to assess whether shear wave elastography could be used to determine EHPSS closure. As a secondary objective, measurements obtained from both intercostal and subxiphoidal views were compared. Mean values for the average, median, and maximum two-dimensional shear wave velocities (2D SWV) for the closed EHPSS were 2.88 +/−0.11 m/s; 2.83 +/−0.11 m/s; and 3.75 +/−0.16 m/s, respectively. In the MAPSS dogs, mean values for the average, median, and maximum 2D SWV were 2.77 +/– 0.17 m/s; 2.71 +/– 0.17 m/s; and 3.66 +/−0.24 m/s, respectively. No significant differences in 2D SWV were present between dogs with closed EHPSS and those with MAPSS (*P* = 0.33; *P* = 0.33; *P* = 0.42, respectively). When assessing potential differences between intercostal and subxiphoidal 2D SWV measurements, no effect was observed for the average and median 2D SWV (*P* = 0.06; *P* = 0.07, respectively). Yet, a significant difference was identified for the maximum 2D SWV between intercostal 4.00 +/−0.20 m/s and subxiphoidal 3.41 +/−0.17 m/s measurements (*P* = 0.02). The relevance of this finding is uncertain as many other studies about liver elastography only report mean and not maximum values.

## Introduction

Portosystemic shunts (PSS) are vascular anomalies that connect the portal vein to the systemic circulation, bypassing the hepatic sinusoids and the liver parenchyma (1). When blood bypasses the liver, trophic factors (particularly insulin and glucagon) are not available to encourage hepatic growth, resulting in poor hepatic development, and altered fat and protein metabolism, hepatic atrophy, and eventually liver failure (2, 3). Liver histology of dogs suffering from congenital PSS demonstrates many changes such as microscopic bile duct proliferation, hypoplasia of portal tributaries, arteriolar proliferation, and smooth muscle hypertrophy, with some dogs also showing evidence of fibrosis (4, 5). In one recent study, some degree of liver fibrosis was present in about 90% of dogs with congenital extrahepatic PSS (EHPSS), with some even categorized as an advanced stage of fibrosis (6). The degree of portal fibrosis has been suggested to increase with advanced age with a resolution of hepatic changes in dogs with closed EHPSSs (4). In the latter study, liver samples collected before and 8 to 272 days (median 48.5 days) after partial ligation of the shunting vessel were compared; however, confirmation of the surgical success of PSS closure was not described.

As shorter life expectancy is reported in dogs where PSS attenuation is not performed, it is important to try to gradually attenuate PSS without causing multiple acquired PSS (MAPSS) to develop (7–11). No ideal non-invasive test exists to discriminate between different surgical outcomes (closed PSS vs. shunt patency through the original PSS or development of MAPPS) (12–15). Computed tomography angiography (CTA), transsplenic portal scintigraphy (TSPS), splenoportovenography, and magnetic resonance imaging (MRI) are considered reliable techniques, but they are invasive, require anesthesia, hospitalization, are high in costs, and are associated with potential morbidity (16–18).

Sonoelastography evaluates the firmness of tissues through which differences between normal and pathological tissue could be made (19, 20). It is a convenient way to visualize, record, and report tissue stiffness parameters. There are four types of US-based elastography techniques available: strain elastography, transient elastography, point-shear wave elastography, and two-dimensional shear-wave elastography (2D SWE)(19, 21). Two-dimensional shear wave elastography uses the acoustic radiation force impulse (ARFI) technique to provide a quantitative assessment of tissue stiffness. The ARFI push generates a transverse (shear) wave that moves slowly in soft tissues and more rapidly in stiffer tissues. Consequently, 2D SWE provides information complementary to conventional ultrasound and is of particular interest due to its non-invasiveness, wide availability, and relatively low cost (21–23).

In human hepatic diseases, elastography has been used primarily to diagnose and monitor the degree of hepatic fibrosis to guide treatment decisions (20) but liver stiffness has also been linked to many other physiological and pathological conditions such as hepatic inflammation, obstructive cholestasis, hepatic congestion, acute toxic hepatitis, amyloidosis, lymphoma, and extramedullary hematopoiesis (20). In recent years, there have been numerous studies pertaining to liver elasticity in dogs and cats (23–31), but no studies describe such modality in dogs with PSS.

The objectives of this study were to compare liver 2D SWE measurements in dogs with successfully closed EHPSS and those with multiple acquired portosystemic shunts (MAPSS) after gradual EHPSS attenuation. Furthermore, the location of the ultrasound probe (intercostal vs. subxiphoidal) for taking the measurements as well as dog characteristics (sex, breed, body weight, and age both at time of surgery and at time of 2D SWE) were analyzed to determine the effect of these values on liver 2D SWE measurements. We hypothesized that patients with MAPPS would have increased tissue stiffness and therefore increased measured velocities. Additionally, we hypothesized that the location of the measurements and dog characteristics would not significantly influence the measured results.

## Materials and methods

### Animals

All procedures were approved by and conducted in accordance with the local ethical and deontological committee (EC 2018-77 and DWZ/ER/1.15/28).

Dogs that underwent gradual surgical treatment for congenital EHPSS in our institution (Faculty of Veterinary Medicine of Ghent University) between 2013 and 2018 were prospectively recruited. To be eligible, the dogs had to have the outcome of surgical attenuation of the EHPSS confirmed *via* TSPS and/or CTA minimally 3 months after the surgical procedure and had to have a postoperative follow-up period of at least 6 months. Signalment, sex, breed, body weight, and age both at the time of surgery and at the time of 2D SWE were recorded at the time of recruitment.

### Measurement of two-dimensional shear wave elastography

**Figure 1:**
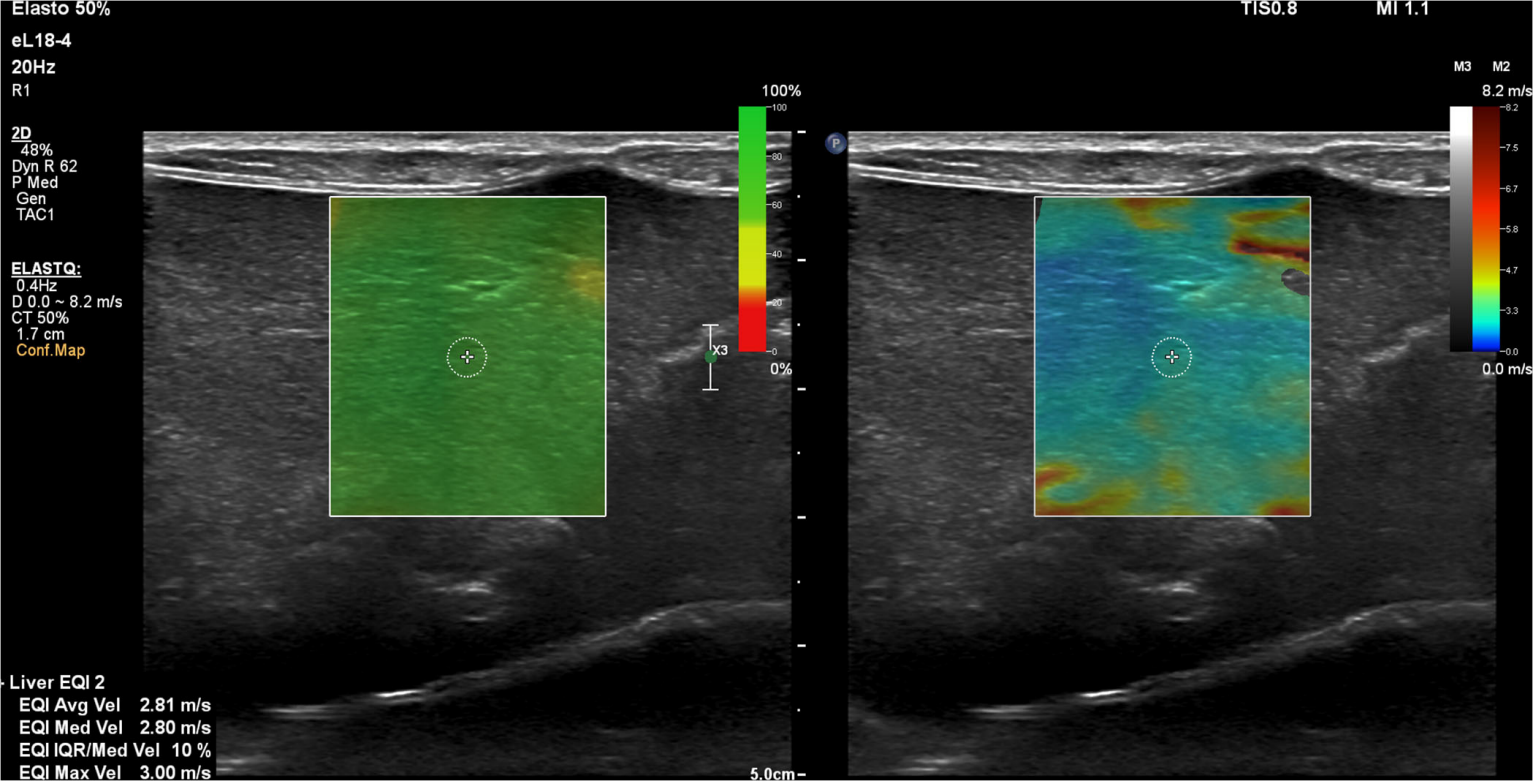
Representative two-dimensional shear wave elastography (2D SWE) image of the right lobe of the liver using the subxiphoidal approach for a speed mode in a dog with a closed extrahepatic portosystemic shunt. The green confidence map and the elastogram image are displayed concurrently over the B-mode image. A small circular shape sample area within the ROI is selected to produce the 2D SWE measurements.

Ultrasound examination was performed between July 2019 and January 2020 by a board-certified veterinary radiologist (E.S). In all the dogs, 2D SWE was performed without sedation using Philips ElastQ Imaging (ElastQ, software version 3.0.3, Philips, Brussels, Belgium) and a linear probe (Pure Wave eL18-4 ultra–broadband linear array transducer, Philips). In accordance with the guidelines for the clinical use of elastography of the liver in humans (20, 21, 32) and the published results in dogs (23, 25, 27, 29–31), the subxiphoidal and left lateral recumbency intercostal approach for acquiring the elastography measurements from the right liver lobe was used. The provided confidence map highlighted areas with optimal shear wave propagation for improved ROI (region of interest) placement. Within the confidence map, every pixel in the ROI is assigned a confidence value from 0 to 100 and a corresponding color between red and green. Low values (red) indicate that the stiffness value for a given pixel is less reliable. High values (green) indicate that the stiffness value for a given pixel is more reliable (Figure 1). The confidence threshold was set at 50%, as recommended by the manufacturer (Philips, Brussels, Belgium), meaning that regions with a confidence value of <50% were rendered transparent and not measurable. Measurements were recorded either in kPa (pressure) or m/s (velocity). For the consistency of measurements, the interquartile range (IQR)/median was set to be <30%. The IQR is the spread of 50% of the measurements around the median and thus the IQR/median is an effective way to assess the quality of the range of measurements (32). Using the visual control of the 2D B-mode image, an artifact-free image of the liver parenchyma was chosen for subsequent SWE measurements. A circular shape sample area within the ROI was set at 5 to 10 mm in diameter and excluded regions that were not color coded. The sample area was positioned in the parenchyma of the liver at least 10 mm deep to the liver capsule and at least 5 to 10 valid measurements were obtained, and the resultant mean 2D SWV was calculated and used as representatives. The scanner software calculates simultaneously the average, median, and maximal 2D SWV in m/s or kPa (Young's modulus). The measurements were recorded and used for further analysis.

### Statistical analysis

Statistical analysis was performed with SAS (SAS V.9.4, SAS institute). Two-dimensional shear wave velocities were compared between dogs with closed EHPSS and those with MAPSS using a mixed model with the animal as random effect and outcome (closed EHPSS vs. MAPPS), and location (sub-xiphoidal vs. intercostal) and their interaction as categorical fixed effect factors.

## Results

### Study population

Fifteen dogs with closed EHPSS and 6 patients with MAPSS met the inclusion criteria. Out of the 21 patients from the initial cohort, one dog from the EHPSS group was excluded due to poor cooperation and inability to acquire good quality 2D SWE images.

Of the 20 dogs included in study, 1 was intact male, 6 were neutered male, and 13 were neutered female. The mean body weight at the time of elastography on the closed EHPSS group was 5.8 kg (+/−0.67), ranging from 2.2 to 9 kg. The mean body weight in dogs with MAPPS was 5.3 kg (+/-0.98), ranging from 2.8 to 9.2 kg. The mean age at the time of surgery in the closed EHPSS group was 31 months (+/−5,6), ranging from 4 to 72 months. The mean age at the time of surgery in the MAPSS group was 16 months (+/−5.2), ranging from 5 to 38 months. The mean age at the time of 2D SWE in the closed EHPSS group was 72 months (+/−7.5) and 62 months (+/−12) in the MAPPS group. The represented breeds were as follows: 3 Chihuahuas, 3 Maltese dogs, 3 Yorkshire Terriers, 2 Bichon Frise dogs, 2 cross breed dogs, 2 Dachshunds, 2 Miniature Schnauzers, and 1 German Spitz, Norwich Terrier, and Pug.

### Comparison of two-dimensional shear wave elastography between closed EHPSS and MAPSS

**Figure 2:**
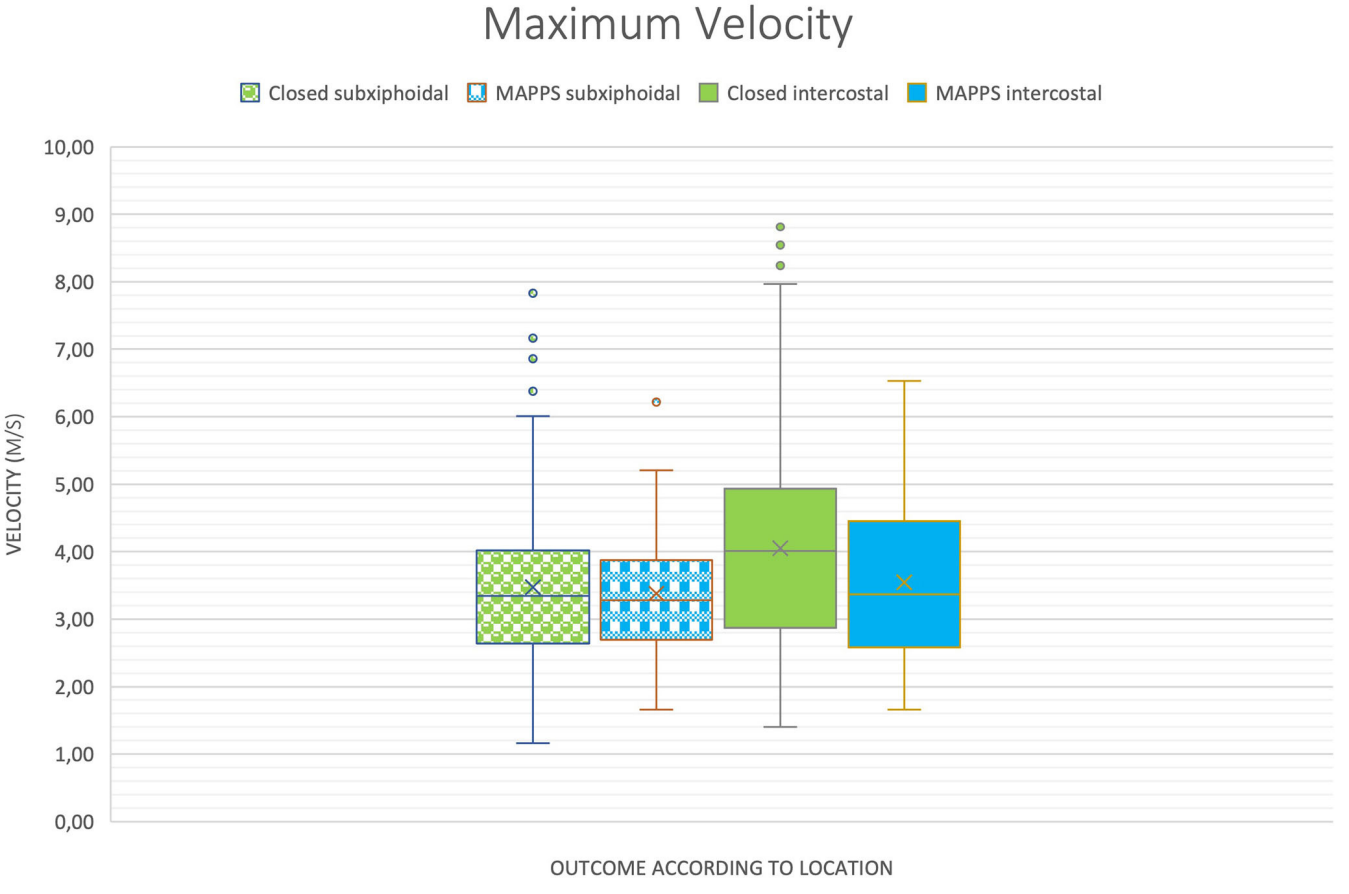
Boxplot of two-dimensional shear wave velocities (2D SWV) depending on location and outcome, demonstrating similar means between the groups, except for maximum shear wave velocity (SWV).

**Figure 3:**
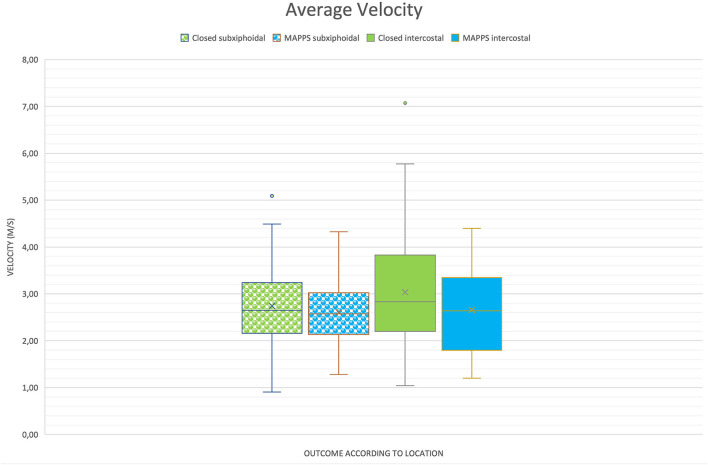
Boxplot of two-dimensional shear wave velocities (2D SWV) depending on location and outcome, demonstrating similar means between the groups.

Results of the study are provided in Table 1 and are illustrated in boxplot diagrams (Figures 2, 3).

**Table 1:** Mean two-dimensional shear wave velocity (2D SWV) of dogs with closed extrahepatic portosystemic shunts (EHPSS) and multiple acquired portosystemic shunts (MAPSS).

**2D SWV**	**Average**	**Median**	**Maximum**
	**(m/s)**	**(m/s)**	**(m/s)**
Closed EHPSS (*n* = 14)	2.88 +/−0.11	2.83 +/−0.11	3.75 +/−0.16
MAPSS (*n* = 6)	2.77 +/−0.17	2.71 +/−0.17	3.66 +/-0.24
*P*-value	0.33	0.33	0.42

No significant differences in 2D SWVs between dogs with closed EHPSS and those with MAPSS were identified (Table 1) regarding average, median, and maximum SWV (*P* = 0.33; *P* = 0.33; *P* = 0.42, respectively).

There was a significant difference between the maximum 2D SWV measurement of the intercostal (4.00 +/−0.20) and the subxiphoidal (3.41 +/−0.17) approach (*P* = 0.02). Regarding the average and median SWVs, the difference was not statistically significant (*P* = 0.06 and *P* = 0.07, respectively).

No significant effects on the average, median, and maximum SWV by sex (*P* = 0.18; *P* = 0.19; *P* = 0.21, respectively), body weight (*P* = 0.32; *P* = 0.35; *P* = 0,24, respectively), age at time of surgery (*P* = 0.53; *P* = 0.53; *P* = 0.45, respectively), or age at time of elastography (*P* = 0.42; *P* = 0.42; *P* = 0.59, respectively) were observed.

## Discussion

The objective of the present study was to compare the long-term postoperative liver stiffness measurements in dogs that underwent gradual surgical attenuation of congenital EHPSS and had successful closure of the EHPSS and those that developed MAPSS. The goal was to determine if non-invasive 2D SWE could potentially predict the success of surgical attenuation. Despite the current literature (8, 10) contradicting the relevance of persistent subclinical postoperative shunting on the life quality of dogs, establishing the surgical outcome would help to better compare survival time, quality of life, and best surgical techniques between these dogs. Many retrospective studies analyzing the long-term clinical outcome of surgical shunt attenuation have presumed shunt closure with no actual post-operative imaging to support that (9, 33). Thus far, CTA has been used as a method for quantifying changes in liver volume and hepatic perfusion after surgery and found to be useful as a quantitative marker of shunt fraction postoperatively (34, 35). Similarly, TSPS can determine the surgical outcome (36, 37). Both imaging techniques allow shunt fractions to be calculated and assessed pre–and post-operatively. Nevertheless, both these modalities require anesthesia and are associated with radiation exposure; therefore, the potential of elastography was investigated in the light of many recent studies demonstrating its usefulness in animals (23, 26, 38, 39).

As portal fibrosis development along with other pathological histomorphological hepatic changes is suggested to be associated with prolonged shunt patency and MAPSS (4–6), it would be expected for MAPPS dogs to have increased liver stiffness which might be demonstrated with non-invasive 2D SWE (2, 33, 39). Tamura et al. in their recent study showed the correlation between liver stiffness and the clinical stage of hepatic fibrosis in dogs (23). However, their velocity measurements overlapped between healthy controls, necroinflammatory hepatic conditions, and those with clinically insignificant liver fibrosis stages, making it only possible to differentiate advanced stages of liver fibrosis.

The mean 2D SWV between dogs with closed EHPSS and those with MAPSS did not differ significantly in our study. Multiple studies detailing MAPPS formation following PSS attenuation have shown that there is a subset of dogs that have the potential for long-term survival and overall favorable clinical outcome (33, 40, 41). The similar results between our two groups might suggest that the persistent shunting *via* MAPPS does not cause sufficient liver fibrosis to alter liver stiffness measurements with 2D SWE significantly. The mean age at the time of 2D SWE in our dogs was similar in both groups (closed EHPSS 72 months vs. 62 months in MAPPS). The mean time from surgery to elastography between the two groups was also similar (closed EHPSS 40.0+/−4.3 months vs. MAPPS 39.6 +/−8.1 months). At the time of surgery, MAPPS dogs were younger in comparison to dogs in the closed EHPSS group (16 vs. 31 months). This could suggest earlier presentation due to more severe clinical signs consequent to the advanced disease process or due to the known association of clinical signs and age of presentation with different shunt morphology types (42). Unfortunately, we do not have comparative pre-operative 2D SWE measurements to corroborate if any dynamic changes between the pre–and post-operative elastography results were present. This would be an interesting area for future research. However, evaluating 2D SWV measurements later in life might be able to demonstrate differences in liver stiffness between these groups as aging itself has been considered a risk factor for the progression of fibrosis, at least in people. In people, it has been suggested that aging increases the susceptibility to liver fibrosis (43, 44).

Our measurements differ significantly from findings obtained in studies performed on healthy dogs (31, 45). In the study by Tamura et al., the mean SWV for the right lobe of the liver in healthy dogs was 1.51 m/s and in the study by Holdsworth et al., the interquartile range for values obtained from the liver at 0 to 2 cm of depth was 1.18 to 1.88 m/s. It is known that the data comparability between the different elastography technologies, system settings, and parameters will vary as many technical factors are not standardized. Several system factors, in particular, shear wave vibration frequency and bandwidth (20) between different commercial systems and equipment, make measurements comparison and data pooling from different studies difficult. Additionally, many other factors such as the examination procedure itself (use of anesthesia, fasting and resting, selection of ROI), breathing, and cardiac motion can cause variability in the measurements (25). An experiment on phantoms between different commercially available systems demonstrated the difference in measurements between machines and observers in the order of 12% (21). It would be interesting to compare the current results with measurements obtained from a control group of healthy dogs using the same ultrasound system.

Shear wave elastography measurements can be affected by body weight, sex, measurement approach, and depth of the measured organ, with normal canine liver, spleen, and kidney measurements being affected by these variables in one study (31). Our study did not see any significant influence of body weight, sex, or age on the liver stiffness results, but considering the congenital nature of the disease and the predilection for small breed dogs (3), our population was homogenous. Regarding the location of the measurements, a statistically significant difference in the subxiphoidal vs. intercostal approach was identified; however, this was only the case for the maximum velocity. In human medicine, the intercostal approach is recommended as the highest intra–and interobserver agreement was obtained through that approach (20). One veterinary study in healthy Beagle dogs also showed a difference in liver elastography measurements according to approach (46), whereas another study with healthy Beagles did not find any correlation (25). Comparison of the outcomes between different studies is hindered as explained before; furthermore, while our method of recording velocities differentiates average, median, and maximum results, it is often not specified how the mean elastographic velocities in other studies are derived.

Our study had some limitations. The sample size was small, especially for dogs with MAPSS, which may have decreased statistical power and led to type II error. Further studies with a larger number of dogs would allow us to possibly show differences between the two populations. This would most likely require a multi-institutional study design; however, as sonoelastographic technical aspects are ultrasound system specific, pooling and comparing data from different institutions would be unreliable. For data uniformity, it would have been better to implement the liver 2D SWE measurements at the same postoperative time point in all the dogs, especially as shunt recanalization 3 and 5 years postoperatively has been described (47) and as age is suggested to be associated with progression of liver fibrosis in people (44).

The severity of hepatic histologic lesions in dogs with congenital PSSs is believed to be related to the degree of shunting and may vary among liver lobes, especially in patients with intrahepatic shunts (3, 5, 48). Another study showed that hepatic lesions were mostly uniformly distributed in dogs suffering from congenital EHPSS (4); however, in three dogs with EHPSS, they found that the left lobes were slightly more affected than the right ones. As all our measurements were performed on the right liver lobe, it is still possible that these measurements are not fully representative of the entire liver parenchyma. Liver histology at the time of 2D SWE performance would have been interesting, as it would allow us to correlate the degree of fibrosis and other histological liver changes to the measured variables. Due to the invasive nature of obtaining a liver biopsy, this was not considered justifiable.

## Conclusion

Real-time 2D SWE was unable to differentiate between dogs with closed EHPSS and those with MAPSS long-term after gradual attenuation of EHPSS. No previous studies have described the liver stiffness measurements in a population of dogs with hepatic vascular anomalies and our results could serve as a baseline and reference for future studies.

## Data availability statement

The raw data supporting the conclusions of this article will be made available by the authors, without undue reservation.

## Ethics statement

The animal study was reviewed and approved by Institutional Animal Care and Use Committee (EC 2018–77) and the deontological committee of the Federal Public Service Health, Food Chain Safety and Environment (DWZ/ER/1.15/28). Written informed consent was obtained from the owners for the participation of their animals in this study.

## Author contributions

Concept and design: ES, MT, HD, ND, and GS. Acquisition of data: ES, MT, and LD. Analysis and interpretation: MT and LD. Revising article for intellectual content: JS, LD, GS, ND, HD, and ES. Drafting the article: MT. Final approval of completed article: JS, LD, GS, ND, HD, ES, and MT. All authors contributed to the article and approved the submitted version.

## Conflict of interest

The authors declare that the research was conducted in the absence of any commercial or financial relationships that could be construed as a potential conflict of interest.

## Publisher's note

All claims expressed in this article are solely those of the authors and do not necessarily represent those of their affiliated organizations, or those of the publisher, the editors and the reviewers. Any product that may be evaluated in this article, or claim that may be made by its manufacturer, is not guaranteed or endorsed by the publisher.
